# The evolution of consumption and its welfare effects

**DOI:** 10.1007/s00191-016-0459-3

**Published:** 2016-05-06

**Authors:** Ulrich Witt

**Affiliations:** 1 Max Planck Institute for Science of Human History, Jena, Germany; 2 Griffith Business School, Griffith University, Gold Coast, QL Australia

**Keywords:** Consumption, Growth, Satiation, Consumer learning, Preference change, A13, D03, D11, D60, O12, Q01

## Abstract

In this paper the evolution of consumption is explained on the basis of a theory that connects preferences over actions to the motivational forces driving actions. More specifically, the hypotheses about what motivates consumption activities draw on insights from biology, behavioral science, and psychology. With secularly rising income, the growing consumption opportunities and the expanding consumption alter the underlying motivational forces and induce a change of preferences. As a consequence, the structure of consumption expenditures is systematically transformed. In the light of this explanation, the paper analyzes the effects of the growth and transformation of consumption on individual welfare. As turns out, the motivations driving the growth of consumption do not necessarily imply that this growth indeed results in welfare increases, particularly when the ability to spend on consumption is already high. Moreover, when preferences change, the measurement of the welfare effects of the growth and transformation of consumption depends on the arbitrary choice of a reference point. This implies an ambiguity that raises further queries about the normative foundations of the ubiquitous calls for continued consumption growth.

## Introduction

For millennia, poverty and starvation have been the fate of the largest part of human kind. “Nature’s parsimony”, as Ricardo once put it, has only been overcome quite recently. Since about two centuries, per capita income and consumption grow exponentially despite the rapidly rising human population (Maddison 2001). In the most advanced economies, already lower-income classes can now enjoy a standard of living that two centuries ago would have resembled a state of affluence. The flip side of the massive growth of consumption is an increasing toll on the natural environment. Both at a local and global scale, climate change, resource depletion, soil and water degradation, species extinction, and many other forms of environmental decay threaten the living conditions of future generations (see, e.g., UNEP 2014). Nonetheless, motivated by the quest for better life, calls for further economic growth still dominate in the political and public debate even in the most prosperous economies. But, leaving aside the social costs for the moment, is it at all likely that the equation “more consumption = better life” which was valid for the past two centuries continues to hold? Do ever higher expenditures indeed assure steady improvements in preference satisfaction irrespective of the level of consumption already reached?

As is well known, canonical text book economics approaches these questions as follows (see, e.g., Mas-Colell et al. 1995). Consumer preferences are assumed to be invariably given, i.e. the possibility of forming new preferences on innovations in goods and services is ignored. What the consumers’ preferences are is not specified. They are only claimed to satisfy some formal properties which imply utility functions that increase monotonously with rising consumption expenditures. It then follows that consumers always attain a higher utility index, i.e. realize a welfare gain, by spending more. The equation “more consumption = better life” is guaranteed to hold. But can such a claim indeed be made without knowing how consumers satisfy what preferences? Moreover, can their preferences be taken as a invariable measuring rod for welfare that is independent of what it is supposed to assess, namely the growing consumption possibilities (see Binder 2010 for a critical reappraisal)?

Doubts regarding the empirical relevance of such a portrayal of economic behavior were raised early on (e.g., Veblen 1909, Sen 1977, Elster 1982). Evidence clearly points to preferences that are neither always consistent nor invariably given. If it is admitted that preferences do change, an inter-temporally consistent measuring rod for welfare may still be logically possible. The necessary proviso is, however, that individual preferences only change in a particular, unidirectional way (von Weizsäcker 2005, Bernheim and Rangel 2009). The relevance of this assumption cannot be assessed, of course, without a richer understanding of the causes and mechanisms underlying the changes of consumer preferences.

In order to advance this understanding a behavioral theory of preferences is needed. The key to such a theory, it will be argued in this paper, lies in the relationships between preferences over actions on the one side and the motivational forces driving actions on the other. From a preference subjectivism point of view, asking what *motivates* consumers to choose specific actions may appear futile, if it can be assumed that the reasons of choice reside in the individuals’ inextricable subjective sphere. Admittedly, subjective idiosyncrasies are likely to result in some irreducible inter-personal variation in consumer behavior. This does not mean, though, that there are no commonly shared motivational forces which exert a systematic influence on the mean behavior in the population. As explained elsewhere (Witt 2001), among others innate needs and drives signify as human universals and provide a basis for a generic analysis of the reasons of choice and, hence, of the individuals’ revealed preferences.

Theories assisting an analysis of human motivation in general and the motivation to consume in particular are interdisciplinary by their nature and refer to the evolutionary bases of human behavior (see Brown and Richerson 2014). To better understand the motivational forces and to develop a richer theory of preferences the present paper draws on hypotheses from several disciplines. Among them are the biology of drives and needs and of behavioral adaptations (Leslie 1996, Staddon 2014a, b), sociobiology (Wilson 1978), evolutionary psychology (Saad 2007), cognitive psychology (Bargh et al. 2010), empirical happiness research (Kahneman et al. 1999), and also the revival of sensory hedonism in economics (Kahneman et al. 1997). On this basis it can be discussed in detail why and how consumption changes systematically with a growing income and what the consequences are for human welfare.

The paper proceeds in three steps. Section 2 presents a brief review of relevant biological, behavioral, and psychological hypotheses. They relate to innate (heritable), learned (conditioned), and cognitive motivational forces. It is discussed how these forces change and whether such changes imply a shift in preferences. Section 3 derives some implications of these hypotheses for explaining the evolution of consumption. It is shown that a crucial role is played on the one hand by multi-level learning processes which affect existing motivations to consume, generate new ones, and thus induce preference changes. On the other hand, the development is characterized by differences in the satiation dynamics across different motivations. Both kinds of motivational changes become manifest and transform consumer behavior as increases in per-capita income raise the ability to spend and to learn new behaviors. Section 4 turns to the welfare-theoretic aspects of the evolution of consumption, i.e. to the question of whether and when the equation “more consumption = better life” holds true. As will turn out, the answer raises doubts about how calling for yet more consumption growth can be justified even in the most prosperous economies. Section 5 presents the conclusions.

## Motivational hypotheses as key for understanding preferences

Originally, motivational hypotheses were a center piece of utilitarian economics. In the characteristic hedonistic interpretation, the reasons for taking actions were explained by the utility derived thereby in terms of enjoying pleasures and/or avoiding pains (both explicated in great detail). 1 Yet, in the later transformations of the utilitarian program, motivational hypotheses fell victim to the belief that by “a purging out of objectionable, and sometimes unnecessary connotations (of the Bentham, Sidgwick, Edgeworth variety) … a much less objectionable doctrine” would result (Samuelson 1947, p.90). The new, “less objectionable” doctrine in question was Samuelson’s own revealed preference theory. Now widely adopted in economic textbooks it can no longer explain what the utility index represents (Glimcher 2015). Correspondingly, the sensory hedonistic theory of welfare characteristic of the Benthamite tradition has given way to a positivist substitute based on a hollow notion of preference satisfaction.

However, Samuelson’s revealed preference theory rests on a very strong concept of “rational” decision making. Nourished by experimental research in decision science, serious doubts have arisen more recently with respect to whether decision makers actually live up to that rationality standard (Kahneman 2003, Ariely 2009). Decision making anomalies and puzzles have caused behavioral economics to question Samuelson’s doctrine and to again start a transformation of the theory of economic behavior (Camerer and Loewenstein 2004). Actual choices are now portrayed as coming about in two very different ways (Loewenstein 2000, Kahneman 2011). On one side there are the cognitive, belief-based choices of actions. They are subject to a number of systematic biases. On the other side, actions can be the result of unconditioned and conditioned response behavior that is much less, if at all, cognitively reflected. (In terms of Kahneman’s distinction between systems of decision-making these are the system 2 and 1, respectively.) Correspondingly, behavior can systematically change over time as a consequence of either cognitive or non-cognitive learning processes.

Behavioral economics is thus much better able to account for the richness and complexity of human decision making and learning. Yet, with few exceptions (e.g. Loewenstein 2004), the question of what drives or motivates economic behavior continues to be left out, and welfare theory remains bound to a hollow preference satisfaction criterion (Burnham et al. 2015). To make progress on this front, the results of motivational research in the neighboring sciences have to be integrated into the behavioral approach to economics. A first important step is to recognize that, in choosing their actions, human decision makers follow different kinds of motivations depending on time and circumstances.

A basic motivational force is constituted by innate needs and drives. As part of the genetic endowment, such needs represent human universals. They are therefore a good starting point for identifying generic features of human preferences, i.e. features that, excepting the genetic variance, are widely shared among humans. A significant feature of these needs is the role that need deprivation plays for motivating action. The more deprived a need is, the stronger is the motivation to take actions that are directed at reducing or removing deprivation. If the need is satiated, deprivation vanishes and so does the motivation to act.

For needs related to the biological metabolism of the body, deprivation can be easily identified by symptoms of physiological imbalance or deficiency. The need for food can serve as example. Feeling hungry motivates organisms to engage in foraging behavior. The foraging motivation vanishes once caloric intake reaches the satiation level – albeit reappears when these calories have been burned off. Similar homoeostatic patterns are present for needs such as for water, sleep, food, physical activity, sex, shelter, and clothing, i.e. protecting the body against pain and cold. Prominent needs not directly related to the biology of the body are those for affection, care, sensory and cognitive stimulation (or “arousal”), positive self-image, and status and social recognition. They are also contingent on an existing state of deprivation. The motivation to act is again directed at reducing or removing deprivation. However, the question of whether and how satiation of the needs is eventually attained is more complex (see below).

Consider an action that is motivated by the attempt to satisfy one or several (possibly differently) deprived needs by means of consuming one or several goods. A particular need may be served by consuming several different goods and/or a combination of them. Further, the consumption of one and the same good may serve not only one, but several needs simultaneously. Goods having this feature may be called “combination goods”. For example, eating something can be motivated by a deprived need for food, more precisely for calories. But eating something, i.e. experiencing varied taste, scent, texture, and other properties of food, can simultaneously also serve the satisfaction of a deprived need for sensory stimulation (arousal). In this sense, food can be a combination good. 2 Similarly, housing expenditures are not only motivated by the need for shelter, but often also by a deprived need for status and social recognition (Frank 2007).

In terms of a utilitarian representation these conditions can be captured as follows. Suppose at the time of making a choice a decision maker conceives of a set of action options which appear to be feasible given the decision makers budget constraint. Each action option *i* in the set consists of consuming a bundle of goods and services *j* = *1*, …, *m* that is described by a vector ***x***_*i*_ = (*x*_*i*1_, …, *x*_*ij*_, …, *x*_*im*_). If ***x***_*i*_ would serve need *h* exclusively, the *partial* utility derived from satisfying need *h* by the corresponding action would be given in the familiar form by

1 $$ {u}^h = {u}^h\left({\boldsymbol{x}}_i\right). $$

Satiability of a need *h* then means that the partial utility function (1) has a maximum – the bliss point. If feasible, satiation is attained by consuming a (not necessarily unique) need-specific vector ${\boldsymbol{x}}_{\overline{h}}$ of goods by which the bliss point with respect to *h* is reached.

However, often the assumption that a consumption activity serves one need exclusively is not satisfied. Some of the *m* goods included in a consumption bundle can be combination goods, i.e. serve to satisfy several needs and other action motivations (such as those discussed below) simultaneously. Consider for the moment a set N of innate needs with elements *h* = 1, …, *n*. The *total* utility *U*_*N*_ derived by their satisfaction through the consumption of the bundle ***x***_*i*_ is therefore determined by the functional

2 $$ {U}_N={U}_{N\ }\left[{u}^1\left({\boldsymbol{x}}_i\right), \dots,\ {u}^n\left({\boldsymbol{x}}_i\right)\right]. $$

i.e. the product or the sum (depending on the specification of the functional) of partial utilities which ***x***_*i*_ generates with respect to each of the *n* needs.

The question is how the vector x* that maximizes *U*_*N*_ subject to the income constraint is determined. 3 Formally, the solution can be found by calculus of variation. However, in animal studies it has been found that the brain of higher animals – not to speak of humans – is capable of generating in an automatic fashion a single, aggregate value for each of the actions in the set of perceived options as long as that set is small enough. This provides the basis for choosing the highest option value automatically. 4 In economic diction, the option values can be interpreted as an automatically generated prediction of total utility associated with the different actions or consumption bundles (Glimcher 2015). Accordingly, *U*_*N*_ gives a measure of the spontaneously emerging motivation for undertaking specific consumption activities in order to satisfy a (set of) deprived innate need(s).

This motivation is subject to two different kinds of systematic change over time. On the one hand this is the satiation dynamics which will be discussed in more detail in the next section. It reduces in a way specific to each single need the motivation to consume contingent on the amount consumed (per period) to serve the need. On the other hand there is a learning dynamics that changes the relative strength of the motivation across different needs as a result of the experienced relative success in obtaining need satisfaction. This works as follows. If the choice of a particular consumption activity indeed reduces the degree of deprivation of the underlying need, the effect is a rewarding experience by the decision maker. Such an event has been demonstrated in behavioral research to be an instance of primary reinforcement. 5 Since there are usually many alternative consumption activities serving different needs in different ways, the motivation to choose among them is adapted by reinforcement learning to the respective relative rewards experienced to result in terms of relative need satisfaction.

Put differently, the motivation to act adapts to the decision maker’s opportunities for obtaining reward by consuming different consumption bundles. These opportunities depend on economic factors such as availability, relative prices, and income, but also on the agents’ ability or comparative advantages in experiencing a reward feeling by some actions rather than others. As a consequence of these differences, consumers tend to “specialize” in many different ways, e.g., as gourmets, computer freaks, opera lovers, bodybuilders, spiritualists, and so on. Indeed, a significant part of the empirically observable adaptations over time in idiosyncratic consumer behavior described, but not explained further, in the literatures as “habit formation” (see Pollak 1970, and 1978), can be attributed to such specialization processes guided by reinforcement learning.

Besides adaptations in the frequency of a set of given actions through reinforcement learning there is yet another behavioral mechanism that is heritable and contributes to behavior adaptations. It is labeled conditioning learning and works as follows (Leslie 1996, Chap. 2.13). Suppose a particular action consisting of consuming good *j* is observed to occur. Suppose further that it results in a rewarding experience so that it is subject to primary reinforcement. Now assume that this action (the consumption of good *j*) happens to coincide with the consumption of another good *k* which is experienced as neither rewarding nor aversive in itself. If the coincidence recurs several times, the rewarding experience resulting from consuming good *j* comes to be associated with the consumption of good *k*. By virtue of this association, consuming good *k* starts to be reinforced as well. In this vein, good *k* emerges as a conditioned or secondary reinforcer.

The correlate of this kind of reinforcer is a new, spontaneously emerging motivational force for which utility is then also automatically predicted. For sake of distinction call it an “acquired want”. 6 In this way, a new, not previously existing preference for the corresponding action is created. It typically involves new or not previously considered goods and services. Hence, the dimensionality of the utility functional (2), defined above for the innate needs in isolation, has to be expanded (see Eq. (3) below). The effect of conditioning learning thus goes beyond mere habit formation. The extension is crucial for explaining how innovations enter and alter consumer preferences and, it will be argued below, for understanding the evolution of consumption.

So far the motivation driving consumer behavior and the learning processes that modify it have been discussed as phenomena occurring at a non-cognitive level. However, a characteristic of human behavior is that a motivation to act and related learning processes can be triggered at the cognitive level as well. First, relating to the above discussion of innate needs it has to be added that there are needs of genuinely cognitive nature such as the need for autonomy (Hagger et al. 2006) and the need for a positive self-image and self-esteem (see, e.g., Gollwitzer and Kirchhof 1998, Pyszczynski et al. 2004). Their motivational force derives from states of deprivation as in the case of the previously discussed needs. The need for a positive self-image is particularly relevant for consumption behavior (Lades 2012a). If one’s self-image is cast in doubt, the cognitive dissonance that arises causes a situation of need deprivation that prompts strong aversive emotions. In order to reduce deprivation, decision makers are often motivated to engage in consumption activities that symbolize their ideal self-image, if no other options for restoring a positive self-image are feasible (Dunning 2007).

Second, a major way in which the motivation to act is modified is cognitive deliberation and insightful learning. They lead to cognitive goal-setting and its motivational correlate, goal-striving (i.e. the deliberate pursuit of the goals and sub-goals whose accomplishment is experienced as rewarding, see Bargh et al. 2010). In the context of consumption behavior, goal-striving can be directed at accomplishing cognitively constructed objectives for their own sake such as the satisfaction of efficiency, safety, or convenience considerations by consuming suitable goods and services. But goal-striving can also be instrumental in the context of means-ends relationships, i.e. when the motivation originates from some “deeper” needs or wants whose pursuit is cognitively controlled. 7 In any case, the motivation to act depends also on the cognitive goal-setting process, on how means-ends relationships are constructed, and on how the agents discount the time factor.

To account for the utility derived from satisfying acquired wants and cognitive goals, the functional depicting total utility has to be extended to *U* = *U*(*U*_*N*_, *U*_*W*_, *U*_*G*_). *U*_*W*_ and *U*_*G*_ are utility functionals constructed analogously to Eq. (2). They represent the utility generated by the satisfaction of acquired wants and the accomplishing of cognitive goals respectively. If the number of acquired wants is given by *w* and the number of cognitive goals by *g*, we get as the extended total utility functional

3 $$ U=U\left[{u}^1\left({\boldsymbol{x}}_i\right), \dots,\ {u}^n\left({\boldsymbol{x}}_i\right),\kern0.5em {u}^{n+1}\left({\boldsymbol{x}}_i\right), \dots,\ {u}^{n+w}\left({\boldsymbol{x}}_i\right),\kern0.5em {u}^{n+w+1}\left({\boldsymbol{x}}_i\right), \dots,\ {u}^{n+w+g}\left({\boldsymbol{x}}_i\right)\right], $$

In its full complexity, utility maximization thus requires finding a vector x* that maximizes the extended utility functional (3).

The analysis is complicated by the fact that goal-setting often leads to contemplating not previously recognized consumption activities including new goods and services so that the perceived choice set is extended. 8 The same holds when, by conditioning processes, associations with new consumption activities are learned. If a consumer innovation *s* (a smart phone, say) is introduced to the market and recognized by the consumer as a choice option, the vector of goods and services is extended to ***x***_*l*_ = (*x*_*l*1_, …, *x*_*lm*_, *x*_*ls*_). As a consequence, the dimensionality of the total utility functional (3) expands accordingly.

## Motivational change and the evolution of consumption: the differential satiation hypothesis

In its process of growth, consumption has been undergoing significant transformations. In order to explain the evolution of consumption, focus in this section is on the reaction which the consumers in an economy show when their income, i.e. their ability to spend, increases. This means that, as usual, the reaction will be explored at the level of population averages. They are described in terms of the income elasticities of consumption expenditures and/or Engel curves. The latter depict the variation in the size of the diverse aggregate household expenditure categories as a function of income. Engel curves come in two versions (see Chai and Moneta 2010). One version focuses on the variation of expenditures on particular consumption categories across different income classes at a given time. The other (longitudinal) version describes the changes of the size of the different expenditure categories when the average household income in the economy rises over time. Engel curves (as well as income elasticities) are descriptive tools. They allow to visualize and classify empirical observed changes of consumption, but they do not allow to explain them.

To fill the theoretical gap that exists here we can draw on the motivational hypotheses outlined in the previous section. The question then is whether and, if so, how income-induced changes of consumer expenditures differ depending on the purpose they serve: to satisfy innate needs, acquired wants, or cognitive goal-striving. Hence, the relevant version of the Engel curve is given by the function

4 $$ {X}_{ht} = {X}_{ht}\left({I}_t\right), $$

where *X*_*ht*_ denotes the expenditures of the households in the economy in a period of time *t* (usually a year) on a category of goods and services whose consumption is motivated by the force *h* = 1, …, *n* + *w* + *z. I*_*t*_ denotes average household income (exclusive of savings). 9

To begin the discussion of how the motivational forces can be conjectured to shape aggregate consumption patterns when rising income enables higher spending consider the innate needs first. Consumers share these needs (with some variation). Their responses can therefore be assumed to be similar and, hence, properly represented by the empirically observable time series of the national averages of the household expenditures. Regarding the average reaction to rising income, two hypotheses can be proposed. One hypothesis relates to the average expenditure corresponding to the bliss points of the consumers’ partial utility function (4). In terms of real prices, they can be expected to correspond to the satiation level. The second hypothesis is that, ${X}_{\overline{h}}$ is not equally rapidly approached for all *n* needs when the expenditures serving them are increased. Let there be two needs *f* (food) and *a* (arousal) and assume that *f* is more rapidly satiable than *a*. This means that the income at which ${X}_{\overline{f}}$ is reached according to Eq. (4) is lower than the income at which *X*_*ā*_ is reached. When income grows, the motivation for additional spending on need *f* levels off earlier than in case of need *a*. Likewise, the income level at which the expenditure or budget share *σ*_*ft*_ = *X*_*ft*_/*I*_*t*_ starts to decrease is lower than the income level at which *σ*_*at*_ start to decrease.

The need for food or, more precisely, calorie intake is indeed a good example of a satiable need. The empirical evidence for a decreasing expenditure share is very robust. 10 Data for a whole century available for the U.S. show that per-capita income has risen in real terms by a factor of 6 between 1901 and 2002. Over the same period, the share of household expenditures on food declines from 42.5 % to 13.1 % (Chao and Utgoff 2006). The shrinking expenditure share notwithstanding, the convergence to the satiation level can, of course, be delayed by the fact that other, less rapidly satiable needs than that for calorie intake may simultaneously motivate the consumption of food. As already mentioned, this may be the need for arousal (or sensory and cognitive stimulation) which, for reasons explained below, is not that rapidly satiable.

Eating snacks at all occasions outside the main meals or having “refreshment” drinks etc. may become a form of entertainment which results in buying more foodstuff (quantity effect). If not simply wasted, the additional food drives up calorie consumption – often even beyond the satiation level for calories with the consequence of a growing body weight. The need for arousal can be conjectured to also contribute to the rapidly growing away-from-home food consumption, particularly when it involves sampling restaurants offering foreign cuisines. Moreover, the need for arousal seems to drive a trend to consuming more refined, exotic, and in any case more expensive, “gourmet” food (quality effect). 11 Another example of a motivation influencing food consumption which also seems to induce a quality effect is cognitive goal striving related to health and life-style considerations.

When, as a result of an increasing satiation, the motivation that previously dominated the growth of consumption levels off, this means that the respective consumer goods industry is confronted with increasing market saturation. The need for calorie intake is a case in point. The food industry typically responds by creating innovations that in some way try to shift the bound at which the consumption motivation is satiated. 12 A frequent strategy aims at triggering a quality effect by appealing to other, less easily satiable motivations than the need for calories. Examples are foodstuffs with new features or combined with additional services aiming to provide additional sensory stimulation or to appeal to cognitive goal striving informed by health and life-style motives. The incidence of quality and quantity effects and product innovation explains in good part why food expenditures, despite their declining share in the household budgets, still increase in absolute terms. In the U.S., for example, the increase from 1901 to 2002 was 46 % calculated in $ of 2002 (Chao and Utgoff 2006).

Why are other motivations underlying consumption behavior less rapidly satiable than homoeostatic biological needs such as the one for calorie intake? Regarding the need for arousal this can be explained as follows. The need is in a state of deprivation whenever the sensory and cognitive system lacks sufficiently strong stimuli. One may think here of the nagging feeling of boredom described by Scitovsky (1981). Owing to such boredom, a motivation to act emerges. It drives consumers to seek out actions which trigger pleasant sensory and/or cognitive stimulation, e.g. “entertaining” consumption activities. (The stimuli are perceived as pleasant if they are neither too strong nor too weak, an assessment contingent on the strength of previous stimulations.) However, the removal of deprivation by such activities is only a transitory episode, because it is subject to a stupefaction effect or, in utilitarian terms, to hedonic adaptation.

Increases in spending temporarily raise the level of arousal. But as the adaptation to this level of stimulation proceeds, deprivation of the need reemerges, and with it the motivation to act. In comparison, in the case of the need for calorie intake, deprivation regularly recurs as well but can be reduced by repeating *the same* consumption activity. In the case of the need of arousal the stupefaction effect prevents this. Rather, consumption activities are required which offer stronger stimuli. They can be obtained by switching to goods and services that are usually more expensive – if a growing income makes such an option feasible. 13

The potential for unceasing growth in expenditures corresponding to the need for arousal was, in fact, already envisioned by Scitovsky (1976). Cognitive and sensory stimulation can be obtained by a multitude of consumption activities, many of which may simultaneously serve other needs and wants as well. Striving to satisfy this innate need can therefore be argued to represent an essential motivational force which underlies overall consumption growth. Its influence is especially prominent, however, for the massive growth of the household budget shares of two expenditure categories able to attend to the need for arousal. One of them is “recreation” (including tourism). 14 The other is “entertainment” (including consumer electronics, communication, and social media). Using the example of the U.S. household data again spending on this category grew from 54 $ per year in 1901 to 828 $ in 2002, i.e. by factor 15, (calculated in $ of 2002), the highest relative increase among all expenditures categories (Chao and Utgoff 2006).

A similar adaptation process is also present in the case of the need for positive self-image. Consumers can temporarily reduce an annoying discrepancy between how they currently perceive themselves and their ideal self-image – i.e. a deprivation of their need for a positive self-image – by engaging in consumption activities symbolic of the ideal individual they would like to be. This is particularly true for consumers who tend to define their ideal self-image in terms of material possessions (Lades 2012a, b, Chap. 5). When, as usual, the ideal is not really attainable by the symbolic consumption activities, the discrepancy between the actual and ideal self-image recurs and triggers a motivation to engage in an intensified symbolic consumption. For economies with a high per-capita income it can be expected that the budget shares of expenditures on goods and services typically serving that need tend to rise over time. Among them are, e.g., jewelry, and other personal accessoires, cosmetics, cosmetic surgery, “fitness” enhancing goods and services, bodybuilding, anti-aging products.

The reason for why the motivation to consume is not rapidly satiable is a different one in the case of the need for status and social recognition. Comparing oneself to others is an innate human tendency. Through this comparison, a state of deprivation can be caused when one’s status is felt deficient relative to the status of those to whom one is compared, or to whom one compares oneself. This is often the case when one feels insufficiently recognized especially by one’s peer group. Actions aiming at a status improvement by which deprivation would be reduced are then usually informed by comparisons with individuals or groups just above oneself in the hierarchy (Frank et al. 2014). In economic diction, the preference for status and social recognition therefore represents a positional preference (Hirsch 1978). 15

In many social environments, personal income would count as a major determinant of relative social status – if income were reliably observable. Since this is rarely the case, proxies for income, such as the life style one can afford, serve to assess the relative size of income. In case of a deprived need for status and social recognition one can therefore try to signal an improved status by engaging in suitable consumption activities. Goods and services one buys in order to signal status have to be visible to, and appreciated by, those one wants to impress (Heffetz 2011). Whether this condition is fulfilled by a particular consumption item is largely a matter of conventions that are specific to groups and strata in society (Witt 2011). Moreover, consumption must be sufficiently exclusive so that the status signal cannot easily be imitated. Buying expensive status symbols not economically feasible for those with lower income is a way of ensuring exclusiveness. Examples are large, i.e. expensive, homes, exclusive furniture, fancy cars, jewelry, luxury wristwatches, expensive clothes, visits to exclusive clubs, bars, restaurants, hotel (Charles et al. 2009).

However, if consumers strive to gain social recognition and status by imitating individuals or groups just above them in the social hierarchy, the exclusiveness and status-differentiating effect of symbolic consumption is continually challenged when income is secularly rising. Ever more consumers can then afford to engage in the consumption activities symbolizing a higher status. The consequence is a status-consumption race that on average just preserves everyone’s relative status position (Hirsch 1978, Frank 1999 and 2011).^16^ Due to this inherently instable situation, the budget share of status-related aggregate consumption expenditures can be expected to rise over time, if per-capita income is growing. Empirical evidence supports this hypothesis, 17 as also the long time series of the U.S. household budget shares shows: housing expenditures rose from 23.3 % in 1901 to 32.8 % in 2002, or (in $ of 2002) from $806 in 1901 to $5344 in 2002.

When the growth of income and of the ability to spend continues, innate needs that cannot rapidly be satiated (in an enduring manner) can thus be expected to increasingly be the drivers of the growth of consumption. This argument has been developed exemplarily here for the needs for status and social recognition, arousal, and a positive self-image. The relative insatiability of such needs causes a massive substitution processes and restructuring of the consumer goods industries as rising income brings expenditures on “basic” needs to the point of satiation. However, the less rapidly, or not at all, satiable needs are not the only drivers of the further growth of consumption and its changing composition. Preference learning through conditioning and cognitive learning as it has been discussed for acquired wants and cognitive goal setting also play a decisive role. Wants and goals that presently exist could eventually be satisfied with rising income. Yet, the fact that new wants and goals are learned over and again prevents this form of consumption motivation from ever vanishing.

The two kinds of learning are a concomitant of the previously described specialization of consumers in particular activities. The more specialization proceeds, the finer are the distinctions between products and services which consumers can make and the more refined their demand becomes. By the same token a willingness to pay is created for differences that non-specialized consumers are unable to appreciate or even to recognize. In this way, consumer specialization generates opportunities for ever new niche markets that would not be viable without substantial consumer expertise and sophisticated preferences.

The consumer goods industries are eager to fill these niches by actively nurturing the consumers’ learning and preference formation processes in order to postpone market saturation. A frequent promotion activity is, for example, the attempt to create by various means an association between newly launched products and primary reinforcing instances. The intention is to induce consumers to acquire a preference for the products by conditioning learning at a non-cognitive level. Another promotion strategy aims at the cognitive level. It consists in highlighting product features such as functionality, efficiency, convenience, flexibility, reliability, or safety which are believed to appeal to the consumers’ means-ends-reflections and in this way to elicit cognitive motivation for a buying decision. 18

## Implications for human welfare: when is more consumption better?

The hypotheses developed in the previous sections offer a basis for analyzing how the evolution of consumption affects human welfare or well-being (terms used synonymously here). The point of departure is again the role played by the motivations underlying consumer behavior. More specifically the question is: do welfare improvements depend on how growing consumption expenditures are motivated? Put differently, does it matter for the welfare assessment what preferences the growing consumption expenditures are intended to satisfy? Questions like these are not part of the agenda of modern welfare economics. Lacking hypotheses about the content of preferences, welfare judgments are derived – following the logic of revealed preference theory – from the assumption that all actions are chosen voluntarily in a rational way. It is inferred, therefore, that if they would not make consumers better off (whatever their preferences are) they would not be chosen. It then follows that the growth of consumption means a welfare improvement or, to put it that way, enables consumers to live a better life.

Under conditions of poverty and starvation (as in some countries still prevalent today) it may stand to reason that being able to consume more indeed amounts to a “better life”. In the high-income economies, however, the reality of consumption has moved far beyond such conditions, and the growing expenditures serve purposes whose welfare-improving effects are not so obvious. Phenomena such as hedonic adaptation, self-image problems, and positional (status) preferences, raise doubts about the welfare-enhancing quality of these choices. In addition, individual preferences may change when the set of known options is growing and/or changing. For assessing the welfare effects of the evolution of consumption it is therefore essential to go beyond a theory based on an unexplained preference order or utility index. A behavioral welfare theory needs to account for the impact of the different motivations and the effects of the corresponding adaptation mechanisms.

To start with, consider first the preferences related to innate needs in isolation. In terms of the model in Section 2, satiability of an innate need *h* means that the partial utility function (1) has a maximum corresponding to a need-specific vector ${\boldsymbol{x}}_{\overline{h}}$ (consumed per period of time). As explained, examples of needs for which a bliss point can comparatively rapidly be reached when consumption grows steadily are those for food, clothes, and shelter. Typically, deprivation of these needs is associated with poverty and starvation. Not accidentally, needs like these have been labeled “basic needs” in development economics, and their satisfaction was considered part of the essential requirements for human existence (Streeten and Burki 1978). Actions implying an increased consumption of goods serving these needs do raise welfare provided the satiation level of the needs has not yet been reached.

For innate needs that cannot rapidly, if at all, be satiated by increasing consumption, the welfare implications depend on the kind of adaptation mechanism that is triggered by raising consumption. A significant case in point is the need for status and social recognition. As discussed, income increases tend to fuel a status-consumption race in the course of which all participants on average just tend to preserve their existing relative status. For that reason, a continued growth of expenditures motivated by status seeking alone (i.e. not including combination goods serving other motivations simultaneously) does not result in increased positional preference satisfaction. A welfare gain being absent, increased status expenditures can be argued to be a waste of resources (Frank 1999, 2011).

The need for arousal is another significant case in point. The reason given above for why continued increases in consumption fail to bring about an enduring satiation effect in this case is a special form of endogenous preference change: hedonic adaptation (see Frederik and Loewenstein 1999 on the latter). To be more specific consider the (now time-indexed) partial utility function (1) specific to actions serving the need for arousal (*h* = *a*). Suppose that at a time *t* < *T* a vector ***x***_*o*_ of goods and services maximizes *u*_*t* <*T*_^*a*^ subject to the income constraint. Assume further that in *t* = *T* income increases so that, everything else being equal, a consumption vector ***x***_*o* ′_ ≠ ***x***_*o*_ now maximizes Eq. (1). It implies higher consumption expenditures and leads to a welfare gain since

5 $$ {u}_{t=T}^a\ \left({\boldsymbol{x}}_{o\prime}\right) > \kern0.5em {u}_{t=T}^a\ \left({\boldsymbol{x}}_o\right). $$

Now let hedonic adaptation develop its full effect until time *t* > *T*. This means that the higher level of arousal that was attained by the income increase is eroded. The partial utility function (1) is shifted downwards. The temporary welfare gain associated with the expanded consumption disappears by and large such that

6 $$ {u}_{t>T}^a\ \left({\boldsymbol{x}}_{o\prime}\right) \approx \kern0.5em {u}_{t=T}^a\ \left({\boldsymbol{x}}_o\right). $$

Because of the stupefaction effect, stronger and usually more expensive stimuli are necessary to (temporarily) bring back an elevated arousal and preference satisfaction. Once further income increases make this feasible, the process starts anew. The comparison of the relationships (5) and (6) shows a time asymmetry in assessing welfare gains from increased consumption that is typical in the presence of hedonic adaptation. Whether or not there is a welfare improvement depends on whether a *pre*-preference-change or a *post*-preference-change perspective is taken.^19^

In the case of preferences changes caused by conditioning learning (acquired wants) and/or cognitive goal setting a welfare analysis in terms of the partial utility function (1) is no longer possible. Instead, the relevant welfare measure is the (now time indexed) total utility functional (3). However, the welfare effects of consumption growth are ambiguous here too. The ambiguity relates not least to consumer innovations which have been argued above to induce preference changes of this kind. While in politics and in the public innovations are generally considered highly desirable nowadays, their actual welfare effects may not support such a view. In order to make this point consider a situation at a time *t* < *T* in which a vector ***x***_*r*_ of goods and services maximizes the utility functional (3). If income were increased by an amount ∆*I* at that time, a consumption vector ***x***_*r* ′_ = (*x*_*r* ′ 1_, …, *x*_*r* ′ *m*_) ≠ ***x***_*r*_ would maximize the functional (3). Everything else being equal, a higher consumption expenditures and a welfare gain would occur because *U*_*t* <*T*_(***x***_*r* ′_) > *U*_*t* <*T*_(***x***_*r*_).

Now assume that an innovative consumer good *s* (like smart phone) is introduced to the market at that time. Since the adaptation of preferences to innovations takes time, the consumer does not immediately acquire a want for *s* and/or make it an object of cognitive goal striving. Let this happen only at time *T*. This means that for *t* ≥ *T* both the vector of goods and services is extended by the component *s* and the utility functional by a corresponding new acquired want or cognitive goal. If it is a new cognitive goal that is added, the total utility functional is thus given by

7 $$ {U}_{t\ge T}=\varphi \left[{u}_{t\ge T}^1\left({\boldsymbol{x}}_l\right), \dots,\ {u}_{t\ge T}^n\left({\boldsymbol{x}}_l\right),\kern0.5em {u}_{t\ge T}^{n+1}\left({\boldsymbol{x}}_l\right), \dots,\ {u}_{t\ge T}^{n+w}\left({\boldsymbol{x}}_l\right),\kern0.5em {u}_{t\ge T}^{n+w+1}\left({\boldsymbol{x}}_l\right), \dots,\ {u}_{t\ge T}^{n+w+g+1}\left({\boldsymbol{x}}_l\right)\right] $$

with ***x***_*l*_ = (*x*_*l*1_, …, *x*_*lm*_, *x*_*ls*_).For the sake of the argument assume that, after the new preferences including *s* have been formed, income indeed happens to increase by the amount ∆*I*. Let the maximization of Eq. (7) subject to the higher income result in the optimal consumption vector x* = (*x*_1_^*^, …, *x*_m_^*^, *x*_s_^*^). Obviously,

8 $$ {U}_{t\ge T}\ \left({\boldsymbol{x}}^{*}\right) > {U}_{t\ge T}\left({\boldsymbol{x}}_{r\prime}\right). $$

This means that from a *post*-preference-change perspective underlying relation (8) the increase in consumption expenditures, including the spending on the innovation, results in a welfare gain. In such a perspective, foregoing the consumption of smart phones, say, would amount to a welfare sacrifice. When considered from a *pre*-preference-change point of view, however, the opposite holds:

9 $$ {U}_{t<T}\ \left({\boldsymbol{x}}^{*}\right) < {U}_{t<T}\left({\boldsymbol{x}}_{r\prime}\right), $$

because an expenditure on something that is not (yet) valued would result in a welfare loss.

Thus, to the extent to which the secular growth of consumption is driven by newly acquired wants and cognitive goal setting, it is an open question whether such growth results in welfare gains. The answer depends on what time perspective is chosen for measuring welfare. The reversal of the inequality sign in relations (8) and (9) means that any of the welfare judgments regarding the growth of consumption is contestable. Consumers in high-income economies usually choose the post-preference-change perspective when making such assessments. This attitude is quite in line with a mindset that emphatically welcomes stimulation through experiencing the novel things which are readily supplied by the consumer goods industries. *Post festum*, the credo “more consumption = better life” always turns true.

However, the ceaseless development of consumer innovations – with their influence on acquired wants and cognitive goal setting – paves the way for further instances of preference change. Consequently, every post-preference-change situation can be equally said to represent a pre-preference-change situation. Under such conditions, the pursuit of enhanced preference satisfaction through expanding consumption becomes a *drift* process. Its direction is contingent on what preferences happen to be learned when. From a normative perspective, the ambiguity in assessing welfare is suggestive of strong preference relativism. The choice of either the post-change or the pre-change preferences as the measuring rod reflects an implicit value judgment. Utilitarian ethics offers no criterion by which it could be decided which one to choose. If, perhaps as a consequence of recognizing the preference relativism, a commitment to measuring welfare from the pre-preference change perspective is made, one can control and constrain one’s cognitive and non-cognitive learning processes.

There are indeed acute reasons for considering such a self-imposed moderation, namely the private and social costs of the way in which consumption evolves and grows. With respect to the private costs, the rising income that fuels expanding consumption is not feasible without increasing effort and strain on the part of the economic agents. In the high-income economies, doubts can be raised as to whether the continued growth of consumption makes the agents happier (see also Helliwell et al. 2013). As mentioned, concerns about being caught in a hedonic treadmill may be justified (see Binswanger 2006). The question is whether the additional consumption is worth the greater effort and strain. This is particularly true also with respect to consuming ever more new goods and services if one recognizes that their appreciation has in many cases only recently been learned.

Concerning the social costs, doubts of whether additional consumption is worth it are even more pronounced. Until now, the evolution of consumption has always entailed the greater exploitation of materials, biomass, energy, atmosphere, fresh water, and space. The ongoing changes to the global climate foreshadow the potential for catastrophic future developments (McNeill 2000). If the social costs caused this way had to fully be privately internalized, this would reduce the welfare gains whatever measuring rod for welfare is chosen. The evolution of consumption would then take a different path. However, even when there were hope for that to happen (which is not the case), an evolution of consumption still following the mantra “more consumption = better life” would be unlikely to be sustainable (see, e.g., the Millennium Ecosystem Assessment 2005). Massive concerns are justified especially given that the developing countries with huge populations are likely to try to follow the lead of high-income countries on their path of consumption growth.

As has frequently been criticized, once preference subjectivism is left behind, the utilitarian focus on individual welfare provides much less of a normative orientation than often believed (see Sartorius 2003, Gowdy 2005, Binder 2010). On the basis of the motivational underpinnings discussed in the preceding sections a more differentiated picture emerges that, in view of the mentioned private and social costs, suggests two questions of normative relevance. Can it be ignored that in the richer countries ever larger parts of the growing consumption are motivated by innate needs which induce growing expenditures but improve preference satisfaction only temporarily or not at all? Can it be ignored that much of the growing consumption is driven by a demand for goods and services that would not be missed, had the preference for them not been learned in the first place?

## Conclusions

Modern microeconomic theory treats preferences as an unexplained “black box”. More recent developments in behavioral economics, including the few contributions to establishing a behavioral welfare theory, have basically left the black box untouched. However, the theoretical lacuna makes it difficult to explain the evolution of consumption and its welfare effects. In this paper it has been suggested to fill the theoretical lacuna by an inquiry into the motivational foundations of economic behavior. To accomplish this, one can draw on well-established research results from biology, behavioral science, and psychology. In this way, the understanding of both the evolution of consumption and the conditions under which the equation “more consumption = better life” holds, can be improved.

As a result of rising income, consumption activities in the developed world have been scaled up over the past century to previously unprecedented levels. At the same time, the composition of consumption has changed substantially as expressed in the household expenditure statistics. By the same token, the consumer goods industries had to massively restructure. The core thesis implied by the motivational foundations advocated here is that the observed changes in consumption can be attributed to two causal mechanisms.

The first mechanism rest on the fact that some of the motivations which drive consumption activities start to unfold and change once the resources available for their satisfaction are growing. These are the motivational forces arising from acquired wants and cognitive goal setting whose transformations have been discussed in detail. New preferences are then formed and the utility function is extended by new arguments. As long as the ability to spend increases, consumption that is motivated in this fashion can grow without bounds. Whether the corresponding growth process is welfare-enhancing has been shown to depend on whether or not the most recent, i.e. the post-change, preferences are used as the measuring rod for welfare. If such a moving measuring rod is used it can be argued, however, that the very idea of a better life is subject to a drift process which, in turn, implies a strong preference relativism.

The second causal mechanism relates to consumption motivations that result from innate needs. When the ability to spend increases, some of them seem to be quite rapidly satiable when the consumption of goods serving them goes up. These needs have been referred to as “basic needs” in development economics. Up to the satiation point, expanding consumption results in welfare gains. However, unlike in the low-income economies, signs pointing to stagnating demand and saturated markets for the relevant products indicate that the level of satiation either has already been reached or is closely approached in the high-income economies.

Other innate needs do not seem as rapidly satiable, at least not in a lasting way. The needs for status and social recognitions and for cognitive and sensory stimulation (arousal) have been discussed exemplarily for this type of needs. In the first case, it can be doubted whether any welfare gain can be expected to result from raising the corresponding expenditures. The status preference is a positional preference, and the status position cannot be improved by raising expenditures as long as everyone engages in a status consumption race. In the second case, increased spending does indeed result in an improved need or preference satisfaction and, hence, a welfare gain. Yet this is only a temporary effect. Due to hedonic adaptation (stupefaction) much or all of the previous welfare gain disappears after a while.

Thus, unlike in low-income countries, the growing consumption in the high-income economies is motivated in part by needs that are difficult to satiate and therefore promise little, if any, welfare gains when expenditures are raised further. In part, consumption growth is driven by newly emerging preferences whose satisfaction does, or does not, result in welfare gains, depending on what state of the preferences is used as measuring rod. Taking a normative perspective, both findings are problematic, given the massive social costs and environmental threats of a continued consumption growth. It does not seem easy under these conditions to provide a normatively convincing legitimization for the calls for having even in the high-income economies ever more consumption growth.

## Footnotes

1 Motivational hypotheses “…describe why a person in a given situation selects one response over another or makes a given response with greater energization or frequency”, Bargh et al. 2010, 286). If, for example, a consumer chooses to buy food, the Benthamite explanation would be that this action is motivated by the expectation of the pleasure of eating and/or the avoidance of the pain of hunger.

2 Combination goods typically possess several characteristics in the sense of Lancaster (1971). However, there is no unique correspondence between characteristics and needs: one characteristic can be relevant for several needs and several characteristics for one need.

3 If the available budget allows to reach the bliss point of need *h*, the motivation to further expand consumption serving need *h* vanishes by definition. However, a consumption bundle may include combinations goods that serve other, less rapidly satiable, consumption motivations as well. In the optimum, these motivations can drive the consumption of a combination good *j* beyond the bliss point of one or some of the involved needs. Put differently, due to the existence of combination goods, maximizing the utility functional (2) can result in ${x}_j^{*}\kern0.5em >\kern0.5em {x}_{j,\ \overline{h}}$ for some *h*.

4 See Shizgal (1999). Automatic here means that the neural processes involved in the generation of the option (or predicted reward) values occur at the non-cognitive level. Although the processes are not yet fully understood, it seems that they are represented by the local dopamine concentrations. The generating processes are strongly context-dependent and may be conditioned by neurochemical influences (Glimcher 2015); see also Schultz (2002) and Daw and Tobler (2013).

5 Staddon and Cerutti (2003). It should be noted, however, that the deprivation of innate needs is not a necessary condition for reinforcement to occur in the behavior of a species.

6 See Witt (2001). To give an example, let good *j* be gyros or any other dishes served in the hotel restaurant while on vacation in Greece. Let good *k* be the Sirtaki music that is continuously played in the restaurant. Assume that for the consumer this music is an entirely new and initially neutral experience. Conditioning learning between eating the dishes and listening to the music can then result in Sirtaki music becoming an acquired want, i.e. a rewarding experience in its own right. For the effect of conditioning learning to be maintained, the association between consuming goods *j* and *k* must at least sporadically be reaffirmed. For that reason, it is possible for acquired wants to be “unlearned”. Despite some common cultural influences, the idiosyncrasies of the individuals’ conditioning histories cause a substantial inter-personal variation with respect to what wants are acquired.

7 Humans can train themselves to delay the rewarding experience of goal attainment, i.e. the ultimate gratification in terms of the need or want satisfaction, until a date far into the future by “mental time traveling” (Suddendorf and Corballis 1997).

8 The proviso is, of course, that the increasing number of alternatives does not make the decision maker forget options she was previously aware of. Cognitive learning processes are always selective with respect to what new consumption possibilities gain attention. For that reason, cognitive learning contributes to consumer specialization.

9 Maximization of Eq. (3) subject to an expanding budget constraint results in individual income-expenditure curves for the goods and services. These curves for the individual consumers could, in principle, be summed over all consumers yielding the aggregate Engel curves. A prerequisite is, however, that the growing income does not result in preference changes through the emergence of new wants and cognitive goals and/or in an expansion of the choice set by consumer innovations. This condition is not met during the evolution of consumption. Instead of a “micro foundation”, i.e. the aggregation step by which Eq. (4) would be derived from Eqs. (3) and (4) is therefore directly based here on the available aggregate household expenditure data.

10 Pooling long term data from the UN National Accounts Statistics for 64 countries, Kaus (2012) estimates cross-country Engel curves for all COICOP (UN classification of individual consumption according to purpose) expenditure categories. The estimate for food expenditures clearly supports the theoretically expected relationship $d{\sigma}_f/dt = \frac{\partial {\sigma}_f}{\partial {I}_t}\frac{d{I}_t}{dt} < 0$ for the budget share *σ*_*f*_ of food.

11 Given that calorie content is an objective measure, food consumption is a good example to illustrate the two effects. Let a composite commodity *j* be the only good that serves the need for calorie intake (a food basket whose elements are consumed in fixed proportions, say). Assume that *x*_*j*_ units, each with calorie content *c*_*j*_, are consumed on average per period (time index suppressed for convenience). The result is an average intake of (i) *q*_*j*_ = *c*_*j*_ *x*_*j*_ calories per period. If *p*_*j*_ denotes the price, the average food expenditure is (ii) *X*_*j*_ = *p*_*j*_ *x*_*j*_. By inserting (i) into (ii) the expenditure satiation level follows as (iii) ${X}_{\overline{j}}=\kern0.5em \frac{p_j}{c_j}\ \overline{q}$, where $\overline{q}$ is the satiation level for calories. However, if the consumption of *j* is also motivated by additional, less rapidly satiable needs, this may lead to ${X}_j > {X}_{\overline{j}}$ when income increases sufficiently, i.e. the quantity effect implying waste of food and/or overeating. The additional motivations may, of course, also induce a higher willingness to pay for a recomposed commodity with similar calorie content but more entertaining features. Since the price per calorie *p*_*j*_/*c*_*j*_ in eq. (iii) represents a degree of freedom, accepting a higher price shifts ${X}_{\overline{j}}$ upwards – the quality effect. For an empirical study of the effects see Manig and Moneta (2014). In elasticity terms, the two effects can be decomposed into ${\eta}_j=\frac{\partial {p}_j}{\partial I}\frac{I}{p_j} + \frac{\partial {x}_j}{\partial I}\frac{I}{x_j}$.

12 In a study of the history of sugar consumption, Ruprecht (2005) shows that the food industry in the U.S. responded in the 1960s to growing dietary health and life style concerns (a concomitant of rising per-capita income) by innovative products. Sugar as high-calorie sweeteners was replaced by artificial, low-calorie sweeteners as, e.g. in the newly introduced Diet Coke. By eq. (iii) in footnote 11 a reduction of *c*_*j*_ shifts ${X}_{\overline{j}}$ upwards.

13 The history of tourism and of the entertainment industry offers much evidence, see Chai (2007). One of his instructive examples is that of the British working class that considered at the end of the 19th century a leisure trip to Blackpool as major source of arousal. To nowadays reach a level of arousal comparable to the one once elicited by a trip to Blackpool, it is necessary to venture as far as the Balearic Islands or so.

14 The Engel curve corresponding to this expenditure category shows the most consistent, monotonous increase among all expenditures categories in the cross-country estimations conducted by Kaus (2012) on the data mentioned in footnote 10.

15 In order to account for positional preferences, the model in Section 2 would have to be extended. Not only the own action and corresponding consumption vector would have to be entered into the individual partial utility function (1) specific to the status need but also those of the comparison group. The individual total utility functional (3) would have to be expanded accordingly.

16 Status-signaling by means of consumption activities conforming to social norms that change in a trickle-down fashion across social strata can also result in delayed or not enduring need satisfaction. See the discussion of “fashion cycles” in Weidlich and Brenner (1995) and Chai et al. (2007) or of personal cleanliness norms slowly winding up in Woersdorfer (2010).

17 Kaus (2012) follows Charles et al. (2009) and identifies status-signaling behavior with the expenditure categories “housing & utilities”, “furnishings & household equipment”, “transport”, and “miscellaneous goods & services”. Based on the data mentioned in footnote 10 he estimates cross-country Engel curves for each of these expenditure categories and finds the hypothesis supported.

18 How consumer learning and supply-side promotion of innovations jointly drive consumption growth and transform an industry has been highlighted by Frenzel Baudisch (2006) in his case study of the U.S. footwear industry. American consumers traditionally owned and, hence, bought relatively few pairs of shoes until in the early 1970s. Correspondingly, until then footwear demand had an income elasticity smaller than one. From that time on, however, the income elasticity turned greater than one. The reason was the unfolding of an innovative, functional diversification of footwear after the monopoly of the United Shoes Machinery Company was broken up in the late 1950s. Starting with the athletic shoes fashion wave new materials and new production methods allowed not only more rapidly changing fashion colors, forms, textures, and design-driven branding (adding status signaling and entertaining features to the function of footwear). The industry also began to massively advertise speciality shoes for each and every different purpose (appealing to cognitive goal striving). U.S. consumers responded by purchasing and owning significantly more shoes per period so that, notwithstanding the fact that the average price fell, footwear expenditures started to grow more rapidly than per-capita income.

19 The contradictory welfare assessments can be argued to point to a hedonic treadmill effect (Binswanger 2006). For an important part of their consumption, consumers make ever greater expenditures without ever getting really happier. A similar argumentation also applies for the need for a positive self-image, but will not be developed here for reasons of lacking space.
